# Qing Hua Chang Yin attenuates lipopolysaccharide-induced inflammatory response in human intestinal cells by inhibiting NF-κB activation

**DOI:** 10.3892/etm.2013.1071

**Published:** 2013-04-22

**Authors:** XIAO KE, JINGTUAN CHEN, XIN ZHANG, WENYI FANG, CHUNBO YANG, JUN PENG, YOUQIN CHEN, THOMAS J. SFERRA

**Affiliations:** 1 Department of Gastroenterology, Second Affiliated Hospital of Fujian University of Traditional Chinese Medicine, Fuzhou, Fujian 350003;; 2 Academy of Integrative Medicine, Fujian University of Traditional Chinese Medicine, Minhou Shangjie, Fuzhou, Fujian 350122, P.R. China;; 3 Rainbow Babies and Children’s Hospital, Case Western Reserve University School of Medicine, Cleveland, OH 44106, USA

**Keywords:** Qing Hua Chang Yin, traditional Chinese medicine, ulcerative colitis, inflammation, NF-κB pathway

## Abstract

Ulcerative colitis (UC) is a major form of inflammatory bowel disease (IBD), which is tightly regulated by the nuclear factor κB (NF-κB) pathway. Thus, the suppression of NF-κB signaling may provide a promising strategy for the treatment of UC. Qing Hua Chang Yin (QHCY) is a traditional Chinese formulation, which has been used for a number of years to clinically treat UC. However, little is known with regard to its anti-inflammatory properties. In the present study, lipopolysaccharide (LPS)-stimulated Caco-2 cells were used as an *in vitro* inflammatory model of the human intestinal epithelium to evaluate the anti-inflammatory effects of QHCY and its underlying molecular mechanisms. We observed that QHCY inhibited the inflammatory response in intestinal epithelial cells as it significantly and concentration-dependently reduced the LPS-induced secretion of pro-inflammatory TNF-α and IL-8 in Caco-2 cells. Furthermore, QHCY treatment inhibited the phosphorylation of IκB and the nuclear translocation of NF-κB in Caco-2 cells in a concentration-dependent manner, indicating that QHCY suppressed the activation of the NF-κB signaling pathway. Collectively, our results suggest that the inhibition of NF-κB-mediated inflammation may constitute a potential mechanism by which QHCY treats UC.

## Introduction

Inflammatory bowel disease (IBD) consists of two main forms: Crohn’s disease (CD) and ulcerative colitis (UC). These conditions are characterized by chronically relapsing disorders of the gastrointestinal tract (1–3). The etiology of IBD is unclear and there are no effective long-term treatments. Current treatment strategies depend on disease severity, and the majority of them are focused on attenuating disease symptoms rather than treating the disease. Furthermore, numerous currently used treatment approaches for IBD may lead to systemic immunosuppression which limits their long-term use. Therefore, there is a need for the development of new therapeutic agents. Complementary and alternative medicines (CAM), particularly herbal therapies, including traditional Chinese medicine (TCM), have received interest as they have relatively few side-effects and have been used as alternative remedies for a variety of diseases, including IBD (4–7).

The inflammatory response is highly regulated by multiple cellular signal transduction pathways, including the nuclear factor κB (NF-κB) pathway, which is capable of being activated by various pathogens, including lipopolysaccharide (LPS). As a main component of the outer membrane of gram-negative bacteria, LPS has been hypothesized to form an important risk factor of IBD (8–10). LPS activates the host Toll-like receptor 4 (TLR4) via direct interaction, which subsequently transduces immune-related signals to the nucleus via transcription factors, including NF-κB (11,12), leading to the positive regulation of the expression of various pro-inflammatory cytokines, including TNF-α, IL-6 and IL-8 (13–19). Therefore, suppression of the NF-κB pathway may provide an effective strategy for the treatment of inflammatory diseases, including IBD.

Qing Hua Chang Yin (QHCY) is a well-known traditional Chinese formulation that consists of a combination of eleven herbs, including *Herba et Gemma Agrimoniae*, *Coptis chinensis Franch*, *Radix Sanguisorbae*, *Radix Paeoniae Rubra*, *Elettaria cardamomum*, *Magnolia officinalis*, *Artemisia capillaris Thunb*, *Herba Eupaatorii Fortunei*, *Semen Coicis*, *Semen Dolichoris Album* and *Poria cocos*. Collectively, these components confer QHCY with the properties of eliminating heat and dampness, as well as strengthening the spleen to nourish vitality (known as tonifying the Spleen Qi in Chinese). Since the accumulation of toxic dampness and heat is one of the major causative factors in the pathogenesis of UC in the TCM system, heat clearing and eliminating dampness provides the basic principle behind the treatment of inflammatory diseases with QHCY. QHCY has long been used in China to clinically treat UC (20–25). However, the precise mechanism of its anti-inflammatory activity remains largely unclear. Using LPS-stimulated Caco-2 cells as an *in vitro* inflammatory model of the human intestinal epithelium, we evaluated the anti-inflammatory effects of QHCY and investigated its underlying molecular mechanisms.

## Materials and methods

### Materials and reagents

Dulbecco’s modified Eagle’s medium (DMEM), fetal bovine serum (FBS), penicillin-streptomycin and trypsin-EDTA were purchased from Invitrogen Life Technologies (Carlsbad, CA, USA). LPS from *Escherichia coli* serotype 055:B5 was purchased from Sigma-Aldrich (St. Louis, MO, USA). Antibodies for Western blot analysis were obtained from Cell Signaling Technology, Inc. (Beverly, MA, USA). All other reagents, unless otherwise stated, were obtained from Sigma Chemicals (St. Louis, MO, USA).

### Preparation of QHCY

In total, 220 g dehydrated *Herba et Gemma Agrimoniae*, 33 g *Coptis chinensis Franch*, 100 g *Radix Sanguisorbae*, 110 g *Radix Paeoniae Rubra*, 56 g *Elettaria cardamomum*, 110 g *Magnolia officinalis*, 110 g *Artemisia capillaris Thunb*, 110 g *Herba Eupaatorii Fortunei*, 220 g *Semen Coicis*, 110 g *Semen Dolichoris Album* and 220 g *Poria cocos* were extracted with boiling water 3 times. The extracts were then combined and concentrated by boiling to a final volume of 1,000 ml. The final concentration of QHCY crude drug was ∼1.4 mg/ml.

### Cell culture

Human colon cancer Caco-2 cells were purchased from the American Type Culture Collection (Rockville, MA, USA). Cells (passages 20–40) were grown in DMEM containing 10% (v/v) FBS, 1,000 mg/l of glucose, 50 U/ml penicillin and 50 *μ*g/ml streptomycin in a 37°C humidified incubator with 5% CO_2_. Cells were subcultured at 85–90% confluence. Caco-2 cells usually reached confluence 3 days after seeding and differentiated into enterocyte-like cells 18–20 days post-confluence. Fully differentiated cells were used for the experiments. On the day of the experiment, the medium was removed and cells were washed twice with DMEM supplemented with 0.5% FBS.

### Enzyme-linked immunosorbent assay (ELISA)

Differentiated Caco-2 cells (20 days post-confluence) in 24-well plates were pre-incubated with various concentrations of QHCY for 1 h prior to being stimulated with 1 *μ*g/ml of LPS for 24 h. At the end of the experiment, the supernatants were collected following centrifugation of the cell culture media at 3,000 × g for 10 min. The secretion levels of cytokines were measured using a human TNF-α ELISA kit (Invitrogen Life Technologies, Camarillo, CA, USA) and a human IL-8 ELISA kit (BD Pharmingen, San Diego, CA, USA), according to the manufacturer’s instructions. All samples were assayed in triplicate. Absorbance was read at 450 nm.

### Evaluation of cell viability by 3-(4,5-dimethylthiazol-2-yl)-2,5-diphenyltetrazolium bromide (MTT) assay

Differentiated Caco-2 cells (20 days post-confluence) in 96-well plates were treated with various concentrations of QHCY for 24 h. The cytotoxic effect of QHCY on the Caco-2 cells was examined using the MTT colorimetric assay. MTT (100 *μ*l, 0.5 mg/ml in PBS) was briefly added to each well and the samples were incubated for an additional 4 h at 37°C. The purple-blue MTT formazan precipitate was dissolved in 100 *μ*l DMSO. The absorbance was measured at 570 nm using a spectrophotometer reader (Model ELX800; BioTek Instruments, Inc., Winooski, VT, USA).

### Western blot analysis

Differentiated Caco-2 cells (20 days post-confluence) in 6-well plates were pre-incubated with various concentrations of QHCY for 1 h prior to being stimulated with 1 *μ*g/ml of LPS for 30 mins. At the end of experiment, the cells were washed with ice-cold phosphate-buffered saline (PBS). The cells were then lysed with cell lysis buffer (50 mM Tris-HCl, pH 7.4, 150 mM NaCl, 0.5% NP-40, 5 mM EDTA, 50 mM NaF and 1 mM PMSF) containing protease and phosphatase inhibitor (PI) cocktails. The cell lysate was centrifuged at 10,000 × g at 4°C for 10 min and the supernatant was collected to examine the protein expression levels of IĸB-α and p-IĸB-α. To determine NF-ĸB nuclear translocation, a nuclear extract was prepared: prior to lysis, the cells were incubated in 0.5 ml hypotonic buffer A (10 mM HEPES, pH 7.9, 0.75 mM spermidine, 0.15 mM spermine, 0.1 mM EDTA and 0.1 mM EGTA) and allowed to swell at 4°C. The cells were then sedimented at 300 × g for 10 min. The supernatant was removed and replaced with 1 ml fresh hypotonic buffer A plus PI cocktail. Cells were homogenized by 10–15 strokes in a Dounce homogenizer and then incubated in 5 ml sucrose restoration buffer which was composed of 0.5 ml hypotonic buffer B (500 mM HEPES, pH 7.9, 7.5 mM spermidine, 1.5 mM spermine, 2 mM EDTA, 2 mM EGTA and 10 mM DTT) and 4.5 ml of 7.5% sucrose). Nuclei were sedimented by centrifugation at 14,000 × g for 1 min. The supernatant was removed and the pellet was resuspended in lysis buffer. Protein concentrations in total lysates or nuclear extracts were quantified using BCA. Equivalent amounts of protein were resolved in 12% sodium dodecyl sulfate-polyacrylamide gel electrophoresis (SDS-PAGE) gels and electroblotted. The PVDF membranes were blocked with 5% skimmed milk and probed with primary antibodies against IĸB-α, p-IĸB-α, p50, RelA and β-actin (1:1,000) overnight at 4°C and then with appropriate HRP-conjugated secondary antibody followed by enhanced chemiluminescence detection.

### Statistical analysis

Data were analyzed using the SPSS package for Windows (Version 11.5; SPSS, Inc., Chicago, IL, USA). Statistical analysis of the data was performed with the Student’s t-test and one-way ANOVA. P<0.05 was considered to indicate a statistically significant result.

## Results

### QHCY inhibits LPS-induced inflammatory response in intestinal epithelial cells

**Figure 1.**
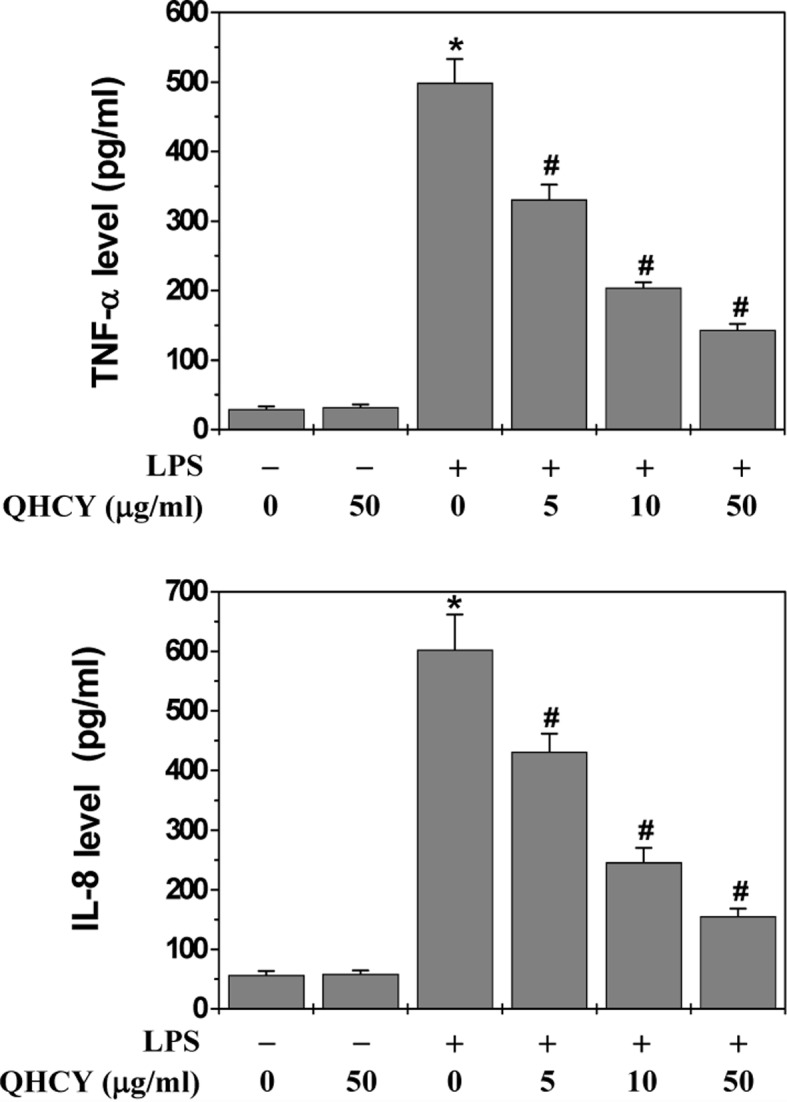
Effect of QHCY on the protein secretion levels of TNF-α and IL-8 in LPS-stimulated Caco-2 cells. Differentiated Caco-2 cells (20 days post-confluence) in 24-well plates were pre-incubated with the indicated concentrations of QHCY for 1 h prior to being stimulated with LPS for 24 h. The secretion levels of TNF-α and IL-8 were examined by ELISA. Data are presented as averages with SD (error bars) from at least three independent experiments. ^*^P<0.05 vs. control cells; ^#^P<0.05 vs. cells treated with LPS but without QHCY. QHCY, Qing Hua Chang Yin; LPS, lipopolysaccharide; ELISA, enzyme-linked immunosorbent assay.

The effect of QHCY on LPS-induced inflammation in differentiated Caco-2 cells was evaluated by measuring the secretion levels of pro-inflammatory cytokines (TNF-α and IL-8) since the release of cytokines is considered to represent an indicator of inflammatory response. Following pretreatment with various concentrations of QHCY for 1 h, differentiated Caco-2 cells were stimulated with 1 *μ*g/ml LPS for 24 h and the levels of TNF-α and IL-8 in the culture supernatant were assessed by ELISA. As shown in Fig. 1, LPS stimulation significantly induced the release of TNF-α and IL-8 in Caco-2 cells. However, QHCY treatment significantly and concentration-dependently reduced the LPS-induced secretion of TNF-α and IL-8, indicating that QHCY may inhibit inflammation in intestinal epithelial cells.

### Cytotoxicity of QHCY in Caco-2 cells

**Figure 2.**
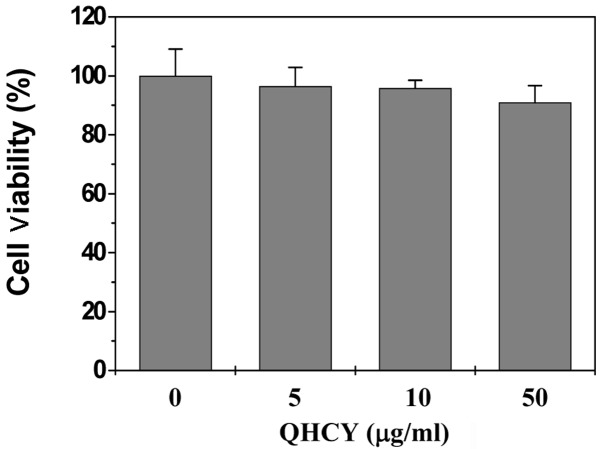
Effect of QHCY on Caco-2 cell viability. Differentiated Caco-2 cells (20 days post-confluence) in 96-well plates were treated with indicated concentrations of QHCY for 24 h. Cell viability was determined using the MTT assay. The data were normalized to the viability of control cells. Data are presented as averages with SD (error bars) from at least three independent experiments. QHCY, Qing Hua Chang Yin; MTT, 3-(4,5-dimethylthiazol-2-yl)-2,5-diphenyltetrazolium bromide.

To exclude the possibility that the anti-inflammatory activity of QHCY was due to cytoxicity, we determined its effect on Caco-2 cell viability using the MTT assay. As shown in Fig. 2, the cell viability was not affected by treatment with QHCY and/or LPS, suggesting that the inhibitory effect of QHCY on LPS-induced inflammation in intestinal epithelial cells did not result from a cytotoxic action.

### QHCY suppresses LPS-induced NF-ĸB activation in intestinal epithelial cells

**Figure 3.**
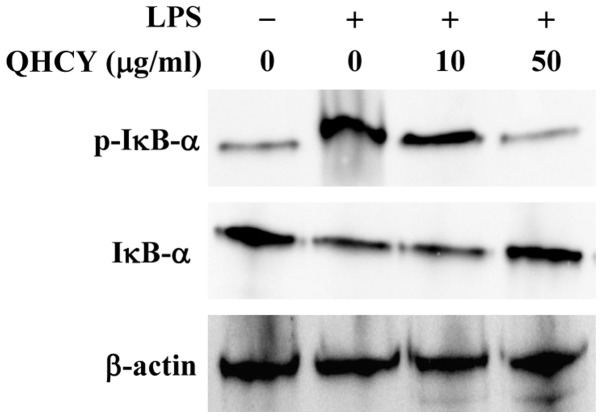
Effect of QHCY on LPS-mediated IκB phosphorylation in Caco-2 cells. Differentiated Caco-2 cells (20 days post-confluence) in 6-well plates were pre-incubated with the indicated concentrations of QHCY for 1 h prior to being stimulated with LPS for 30 min. IκB phosphorylation was determined by Western blotting. β-actin was used as the internal control. Images are representatives of three independent experiments. QHCY, Qing Hua Chang Yin; LPS, lipopolysaccharide.

NF-κB is a critical transcription factor for inflammation response. Activation of the NF-κB pathway consists of several key processes, including the phosphorylation and degradation of IκB and the subsequent nuclear translocation of NF-κB. To investigate the mechanism of QHCY’s anti-inflammatory activity, we examined its effect on LPS-induced activation of the NF-κB pathway in intestinal epithelial cells. Differentiated Caco-2 cells were pretreated with QHCY for 1 h followed by stimulation with LPS for another 30 min, and IκB phosphorylation was examined by Western blotting. As shown in Fig. 3, upon LPS stimulation, the phosphorylation level of IκB markedly increased and this increase was significantly attenuated by QHCY in a concentration-dependent manner.

**Figure 4.**
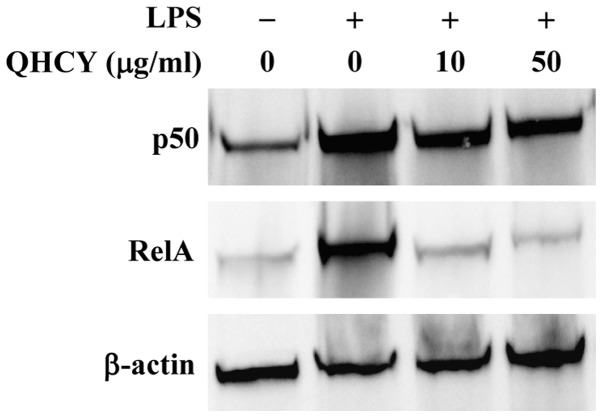
Effect of QHCY on LPS-mediated NF-κB nuclear translocation in Caco-2 cells. Differentiated Caco-2 cells (20 days post-confluence) in 6-well plates were pre-incubated with indicated concentrations of QHCY for 1 h prior to being stimulated with LPS for 30 mins. Nuclear extracts were prepared as described in Materials and methods. The nuclear content of two subunits of NF-κB, p50 and RelA, was determined by Western blotting. β-actin was used as the internal control. Images are representatives of three independent experiments. QHCY, Qing Hua Chang Yin; LPS, lipopolysaccharide.

To verify these results, we examined the change in nuclear content of two subunits of NF-κB, p50 and RelA, to evaluate the effect of QHCY on LPS-induced NF-κB nuclear translocation, which is an important step for NF-κB activation. In agreement with the observed inhibitory effect on IĸB-α degradation, QHCY concentration-dependently prevented the LPS-induced nuclear translocation of p50 and RelA (Fig. 4).

## Discussion

As a major form of IBD, UC is the result of a chronic intestinal inflammatory response (1–3). Since the precise etiology of UC remains unknown, there are no effective long-term treatments. In addition, many currently used UC treatments lead to the development of systemic immunosuppression. Therefore, there is an urgent need for the development of new therapeutic agents. Natural products, including TCM, have received interest as they have relatively few side-effects and have been used as alternative remedies for a variety of diseases, including IBD (4–7). QHCY is a TCM formulation that has been demonstrated to be effective in China for the clinical treatment of UC (20–25). However, the molecular mechanism of its anti-inflammatory activity remains to be elucidated. Therefore, prior to the development of QHCY as an anti-UC agent, the mode of its anti-inflammatory action requires elucidation.

Pro-inflammatory cytokines produced in the intestine, including IL-8 and TNF-α, are important in the pathogenesis of IBD; the release of pro-inflammatory cytokines is therefore considered to represent an indicator of the inflammatory response. Using LPS-stimulated Caco-2 cells as an *in vitro* inflammatory model of the human intestinal epithelium, we observed that QHCY significantly and concentration-dependently reduced the LPS-induced secretion of TNF-α and IL-8, demonstrating that QHCY inhibited the inflammatory response in intestinal epithelial cells. The inflammatory response is tightly regulated by TLRs, a family of pattern-recognition receptors (PRRs), which enable immune systems to recognize pathogen-associated molecular patterns (PAMPs). Different TLRs recognize different PAMPs, including LPS that functions as a specific ligand for TLR4 (8–10,26–29). Following activation by ligand binding, TLR4 transduces the immune-related signals to the nucleus via transcription factors, including nuclear factor κB. As one of the most significant nuclear transcription factors, NF-κB is involved in the control of several important physiological processes, particularly the immune and inflammatory responses. In unstimulated cells, NF-κB is sequestered in the cytosol via interaction with inhibitory IκB proteins. However, when cells receive pathological stimuli, IκB proteins are phosphorylated by IκB kinase (IKK). Phosphorylation of IκB proteins results in their ubiquitination and degradation, which subsequently releases sequestered NF-κB, leading to its translocation to the nucleus where it induces the expression of various pro-inflammatory cytokines (15–19). Using Western blotting, we observed that QHCY treatment inhibited the phosphorylation of IκB and the nuclear translocation of NF-κB in Caco-2 cells in a concentration-dependent manner, suggesting that QHCY suppresses the activation of the NF-κB signaling pathway.

In conclusion, in the present study we demonstrated that QHCY ameliorates the inflammatory response by inhibiting the activation of the NF-κB pathway. Our results further suggest that QHCY may be an effective traditional Chinese formulation for the treatment of UC and other inflammatory conditions.

## Abbreviations:

QHCY: Qing Hua Chang Yin
UC: ulcerative colitis
IBD: inflammatory bowel disease
NF-κB: nuclear factor κB
TLR4: Toll-like receptor 4
LPS: lipopolysaccharide
TCM: traditional Chinese medicine
